# Cannabinoid receptor type 2 agonist JWH-133 decreases cathepsin B secretion and neurotoxicity from HIV-infected macrophages

**DOI:** 10.1038/s41598-021-03896-3

**Published:** 2022-01-07

**Authors:** Lester J. Rosario-Rodríguez, Yamil Gerena, Luis A. García-Requena, Luz J. Cartagena-Isern, Juan C. Cuadrado-Ruiz, Gabriel Borges-Vélez, Loyda M. Meléndez

**Affiliations:** 1 Department of Microbiology and Medical Zoology, School of Medicine, Medical Sciences Campus, University of Puerto Rico, San Juan, PR 00935 USA; 2 Department of Pharmacology, University of Puerto Rico, Medical Sciences Campus, San Juan, PR USA; 3 Department of Biology, University of Puerto Rico, Río Piedras Campus, Río Piedras, PR USA; 4 Department of Chemistry, University of Puerto Rico, Río Piedras Campus, Río Piedras, PR USA; 5 Department of Biology, University of Puerto Rico, Bayamón Campus, Bayamón, PR USA

**Keywords:** Retrovirus, Viral pathogenesis, Cell death in the nervous system, Dementia, Neuroimmunology

## Abstract

HIV-associated neurocognitive disorders (HAND) are prevalent despite combined antiretroviral therapy (cART), affecting 52% of people living with HIV. Our laboratory has demonstrated increased expression of cathepsin B (CATB) in postmortem brain tissue with HAND. Increased secretion of CATB from in vitro HIV-infected monocyte-derived macrophages (MDM) induces neurotoxicity. Activation of cannabinoid receptor type 2 (CB2R) inhibits HIV-1 replication in macrophages and the neurotoxicity induced by viral proteins. However, it is unknown if CB2R agonists affect CATB secretion and neurotoxicity in HIV-infected MDM. *We hypothesized that HIV-infected MDM exposed to CB2R agonists decrease CATB secretion and neurotoxicity.* Primary MDM were inoculated with HIV-1_ADA_ and treated with selective CB2R agonists JWH-133 and HU-308. HIV-1 p24 and CATB levels were determined from supernatants using ELISA. MDM were pre-treated with a selective CB2R antagonist SR144528 before JWH-133 treatment to determine if CB2R activation is responsible for the effects. Neuronal apoptosis was assessed using a TUNEL assay. Results show that both agonists reduce HIV-1 replication and CATB secretion from MDM in a time and dose-dependent manner and that CB2R activation is responsible for these effects. Finally, JWH-133 decreased HIV/MDM-CATB induced neuronal apoptosis. Our results suggest that agonists of CB2R represent a potential therapeutic strategy against HIV/MDM-induced neurotoxicity.

## Introduction

Neurocognitive disorders in HIV-positive people are still prevalent in the cART era, affecting approximately 52% of this population^1^. The different manifestations of HAND include asymptomatic neurocognitive impairment (ANI), mild neurocognitive disorder (MND), and its more severe form, HIV-associated dementia (HAD). After the cART era, the number of HAD cases have decreased, but milder forms of HAND (MND and ANI) are still present^1^. However, the mechanisms by which cART-treated patients still develop these disorders are not completely understood. Immune cells such as macrophages play a major role in the neuroinflammation promoted by HIV infection^2–4^. Our laboratory has found increased expression of a pro-inflammatory lysosomal enzyme, cathepsin B (CATB), in postmortem brain tissue with HIV encephalitis (HIVE) and HAND^5^. CATB is also increased in plasma and monocytes of HIV-positive patients with HAND^6^. Increased secretion of CATB from in vitro HIV-infected MDM and microglia leads to neuronal apoptosis, and it can be prevented by the specific CATB inhibitor CA074 or an antibody^5,7–11^. After HIV infection of MDM, CATB increases its interactions with serum amyloid P component (SAPC) in supernatants, promoting neuronal death^8^. Recently, we reported that HIV-1 infection triggers CATB entry into neurons, cleavage of caspase 3, and decreased expression of synaptophysin, promoting neuronal dysfunction and apoptosis^12^. This effect was reversed by pre-treatment with antibodies against CATB or SAPC. In search of mechanisms of CATB-induced neurotoxicity using quantitative proteomics, we recently found that HIV/MDM-derived CATB and SAPC complex trigger apoptosis in neurons using common mechanisms, which include Tubulin 1A and nuclear Lamin A protein expression deregulation (Zenon et al., in press)^13^. Lamin A is exclusively cleaved by effector caspase-6 to induce apoptosis^14–16^. Therefore, CATB appears to induce apoptosis by affecting the nuclear membrane, and this was reverted by the addition of a CATB antibody. These results might indicate that CATB induces neuronal apoptosis through a caspase-dependent mechanism. Thus, targeting CATB and/or SAPC represents a potential therapeutic strategy against HAND that should be evaluated in future in vivo studies.

New potential treatments that ameliorate the neuroinflammatory responses in HAND are urgently needed. CB2R agonists have been proposed as potential therapeutic strategies against HAND^17–19^. CB2R mediates anti-inflammatory responses in the human brain^20^. This receptor is abundant in the periphery; mostly in immune cells such as macrophages and B cells^21^, and does not mediate the psychoactive effects of cannabinoids^22^. Moreover, some studies have shown increased expression of CB2R in in vitro HIV-infected macrophages and microglia and in postmortem brain tissues of HIVE and HAND patients^23,24^. Similarly, simian immunodeficiency virus (SIV) infection upregulates CB2R expression in T cells, brain resident macrophages, and microglia^25^. CB2R agonist Gp1a reduced neuroinflammatory markers in a mouse model of neuroAIDS^26^. CB2R activation inhibits INFγ-induced microglial activation^27^. Activation of CB2R inhibits migration of macrophages and microglia to the chemoattractant HIV-1 Tat and inhibits Tat-mediated adhesion of monocytes to the extracellular matrix proteins^28–30^. CB2R agonists prevent leukocyte infiltration into the brain and protect the blood–brain barrier integrity^26,31–33^. Activation of CB2R inhibits HIV-1 replication in macrophages, microglia, and CD4 + T cells^24,34–36^. CB2R activation reduces neurotoxicity to HIV-1 viral proteins Tat and gp120^37–41^. Therefore, CB2R represents a promising target for new treatments against HAND^17–19,42^. However, the role of CB2R agonists in HIV/MDM-induced neuronal death and CATB neurotoxic potential has not been previously studied. *We hypothesized that HIV-infected MDM exposed to CB2R agonists decrease CATB secretion and neurotoxicity.* Our results indicate that CB2R agonist JWH-133 reduces CATB secretion and neurotoxicity induced by HIV-infected macrophages.

## Materials and methods

### Cell culture, HIV-1_ADA_ infection, and treatments

Peripheral blood mononuclear cells (PBMCs) were isolated from healthy women blood donors as part of the NIH/NIGMS SC1 project: “Targeting monocyte/macrophage cathepsin B interactome in HIV-1 neurocognitive disorders”, with approval from the University of Puerto Rico-Medical Sciences Campus Institutional Review Board, Human Research Subjects Protection Office (Protocol #0720116). Written informed consents were obtained from all participants. All the experiments were conducted following institutional guidelines and regulations. The rationale for selecting women donors is that HIV-infected MDM from women secrete higher levels of CATB compared to men (Supplementary Fig. 1). PBMCs were cultured, and MDM were isolated by adherence after 7 days of culture in RPMI supplemented with 20% fetal bovine serum (FBS), 10% human serum, and 100U/mL pen/strep (Sigma; St Louis, MO). On day 7 of culture, MDM were infected with the CCR5-tropic HIV-1_ADA_ stock (University of Nebraska). HIV-1_ADA_ strain was originally isolated from PBMCs from a patient with AIDS^43^. HIV-1_ADA_ stock was diluted to a multiplicity of infection (MOI) of 0.1 in serum-free RPMI, and cells were incubated overnight at 37 °C, 5% CO_2_. On the next day, supernatants were removed, cells were washed twice with serum-free RPMI, and treatments were prepared in fresh MDM media and added to cultures, as described below.

For CB2R ligands (JWH-133, HU-308, and SR144528) dose-response curves experiments, MDM were cultured in 6-well plates at a concentration of 5 × 10^6^ cells/well or in 24-well plates (in duplicates or single-well) at a concentration of 2.5 × 10^6^ cells/well. After infection and removal of residual virus, cultures were treated with CB2R agonists, JWH-133 or HU-308 (Tocris), at five different concentrations (0.1, 0.5, 1, 5, and 10 µM). These ligands were selected based on a previous study that demonstrated that they are the most selective and have the least off-target effects for studying CB2R activation^44^; and the concentrations were selected based on previous studies that assessed the effect of JWH-133 in HIV-1 replication in primary MDM at doses ranging from 0.1 to 10 µM^24,36^. Controls included vehicle-treated cells (DMSO: Hybri-Max™ sterile-filtered) (Sigma; St. Louis, MO) and untreated cells. CB2R ligands were reconstituted in 100% DMSO at the maximum solubility recommended by the company (JWH-133: 50 mM; HU-308: 100 mM; and SR144528: 100 mM). From the stock reconstitution, serial dilutions in DMSO were prepared, aliquoted, and saved at − 20 °C as follows: For JWH-133, aliquots of 50 mM, 25 mM, 5 mM, 2.5 mM, and 0.5 mM were prepared for the treatments of 10 µM, 5 µM, 1 µM, 0.5 µM, and 0.1 µM, respectively, to maintain the same volume of treatment at each condition; For HU-308 and SR144528, aliquots of 100 mM, 50 mM, 10 mM, 5 mM, and 1 mM were prepared for the treatments of 10 µM, 5 µM, 1 µM, 0.5 µM, and 0.1 µM, respectively. Treatments were maintained until 12 days post-infection (dpi), removing half of the media every 3 days and replacing it with fresh half media containing treatment. Supernatants were collected and saved at − 80 °C to measure HIV-1 p24 and total CATB levels by ELISA**.** On day 12pi, media was removed and saved at − 80 °C. Cells were washed twice with PBS 1X to remove traces of serum and treatments, and cells were incubated with serum-free RPMI for 24 h at 37 °C, 5% CO_2_. On day 13pi, MDM supernatants (for now on referred to as macrophage-conditioned media or MCM) were collected and saved at − 80 °C for the neuronal apoptosis assay. Cell viability was assessed in MDM cultures at day 13pi. For the antagonist/agonist co-administration experiments, MDM were cultured in 24-wells plates at a concentration of 2.5 × 10^6^ cells/well. After infection and removal of residual virus, cells were pre-treated with CB2R antagonist SR144528 (Tocris) at 1 µM for 1 h, followed by JWH-133 (Axon Medchem) treatment at 0.5 µM. Treatments were maintained, and the co-administration was repeated at day 3pi, replacing half of the media and maintaining both treatments in cultures until day 6pi. Supernatants from 3 and 6dpi were collected and saved at − 80 °C for determination of HIV-1 p24 and CATB levels. The concentration of 1 µM of SR144528 was selected because, in previous dose-dependent studies, we determined that this was the maximum concentration with no effect on HIV-1 replication (data not shown), CATB secretion (data not shown), and cell viability (Supplementary Fig. 2).

For the analysis of surface CB2R levels by Flow cytometry, we cultured PBMCs in non-tissue culture treated T75 flasks at 30 × 10^6^ cells/flask. After infection and removal of residual virus, cultures were maintained in plain MDM media (untreated) until day 12pi, changing media every three days. MDM supernatants were collected for the determination of productive HIV-1 replication using HIV-1 p24 ELISA. For the analysis of surface CB2R by immunocytochemistry/immunofluorescence, MDM were cultured in 8-well Permanox® chamber slides (Thermo Fisher Scientific) at a concentration of 2 × 10^5^ cells/ well. After infection and removal of residual virus, MDM were treated with JWH-133 at 0.5 µM. Treatments were maintained until day 12pi, exchanging media every three days. Slides from days 3, 6, 9, and 12dpi were washed twice with PBS 1X and fixed with 4% paraformaldehyde (PFA) in PBS 1X. For the intracellular CB2R expression experiment, uninfected and HIV-infected MDM were cultured in T25 flasks at a concentration of 10 × 10^6^ cells/well. MDM were cultured for 12dpi, exchanging and collecting media every three days. HIV-1 p24 levels were measured from MDM supernatants, and whole-cell lysates were collected and saved at − 80 °C for Western Blot analyses. For cell viability experiments, MDM were cultured in 96-well plates in triplicates/condition at a concentration of 2.5 × 10^5^/well. After infection and removal of residual virus, MDM were treated with CB2R ligands as described above. At the end of cultures (13dpi), cell viability was measured using an MTT assay. For the neuronal apoptosis assay, human neuroblastoma cells (HTB-11; ATCC) were grown as previously described by our laboratory^5^. In summary, HTB-11 were plated in 8-well glass chamber slides at 2 × 10^5^ cells/well and cultured in Eagle’s MEM (EMEM) with 10% FBS, 1% sodium pyruvate and 1% non-essential amino acids. HTB-11 neurons were incubated at 37 °C, 5% CO_2_ for 3–5 days until 75–80% confluence was achieved.

### Cell viability

MDM viability was assessed at day 13pi, as previously described by our laboratory^9^. In summary, we used 3-(4,5-dimethylthiazol-2-yl)-2,5-diphenyltetrazolium bromide (MTT) assay (Sigma; St Louis, MO), following manufacturer instructions. PBMCs were cultured in 96-well plates at 2.5 × 10^5^ cells/well in triplicates/condition. Metabolically active cells reduce MTT due to the action of dehydrogenases, generating NADH and NADPH reducing equivalents. As a result, an intracellular purple formazan is produced, and it was measured by photometry using Varioskan Flash (Thermo Fisher Scientific). As a positive control, at day 12pi, we incubated MDM with 1% Triton X-100 in serum-free RPMI for 24 h, and we measured cell viability at day 13pi.

### Surface CB2R staining for flow cytometry

We analyzed surface CB2 receptor levels in MDM at day 12pi, which was the last day of exposure to the CB2R ligands. On day 12pi, media was collected and saved at − 80 °C, and MDM were washed twice with PBS 1X. MDM were detached by incubating them with 10 mL accutase solution (Sigma) for 30 min at 37 °C, 5% CO_2_. Then, 10 mL of MDM media were added to each flask to neutralize the accutase. The detached MDM were transferred to a 50-mL tube, counted, and cell viability was calculated using Turks and Trypan Blue dyes. Then, 5 × 10^5^ MDM were transferred to round-bottom 5-mL polystyrene tubes (Corning Science, MX) and washed twice with PBS 1X to remove media. MDM were incubated with an anti-human CB2R-Alexa Fluor® 647 conjugated antibody (R&D Systems) (1:100 in PBS 1X) for 1 h at 4 °C. MDM were washed three times with PBS 1X and fixed with paraformaldehyde (PFA) (0.5% in PBS 1X). Surface CB2R levels were quantified and analyzed using Flow Cytometry.

### Flow cytometry

Flow cytometry analyses were performed using a FACSAria cytometer (BD Biosciences, CA). For data acquisition and multivariate analysis, we used FACSDiva and FlowJo software (BD Biosciences, CA). MDM were first identified and gated in forward/side scatter dot plots. The fluorescence emission for CB2R-Alexa Fluor® 647 antibody was detected in the FL4 photomultiplier through a 670/30 nm bandpass filter. Log mode was used to acquire data on scatter parameters and histograms. We acquired ten thousand events for each sample tube. To quantify surface levels of CB2R, we used the median peak channel from the histograms.

### Immunocytochemistry/Immunofluorescence of surface CB2R

Slides of fixed MDM from days 3, 6, 9, and 12dpi were washed twice with PBS 1X and incubated with blocking buffer (1% BSA and 1% goat serum in PBS 1X) for 1 h in a humidity chamber, shaking at RT. MDM were incubated with a rabbit polyclonal antibody raised against the N-terminal region of human CB2R (1:100; Cayman Chemical) in blocking buffer overnight, shaking at 4ºC. The next day, cells were washed five times with Tris-buffered saline (TBS 1X) and incubated with a goat anti-rabbit Alexa Fluor® 546 secondary antibody (1:500 Thermo Fisher Scientific) in a blocking buffer for 1 h in a dark humidity chamber, shaking at RT. Cells were washed five times with TBS 1X and counterstained with VECTASHIELD® Antifade Mounting Media with DAPI (Vector Laboratories). Fluorescence microscopy was performed using a Nikon Eclipse E400, with camera SPOT Insight QE and Fluorescence X-cite Series 120 under an excitation wavelength of 546 nm and 405 nm for CB2R and nuclei, respectively. The magnification was set at 20X. At least 3 different random pictures per condition were acquired. The mean fluorescence intensity (MFI) values of CB2R were acquired using the NIS Elements Analysis software (Nikon).

### Western blot

Western Blot analyses were performed as previously described by our laboratory^12^, with the following specifications. In summary, the total protein concentration of whole-cell lysates was measured by detergent compatible protein concentration assay (DC; Bio-Rad, Hercules, California, USA), following manufacturer instructions. Fifteen micrograms of intracellular proteins from MDM lysates were diluted with Laemli sample buffer and applied to each well of 4–20% TGX Ready Gel 15-wells (Bio-Rad, Hercules, CA), and transferred to 0.45 PVDF membranes (Bio-Rad). Membranes were blocked with 5% milk in TBS 1X and incubated with rabbit anti-human CB2R (1:200; Cayman Chemical) and a rabbit polyclonal anti-glyceraldehyde 3- phosphate dehydrogenase (GAPDH; 1: 250; BIOSS, Woburn, Massachusetts, USA) shaking overnight at 4 °C, followed by a goat anti-rabbit secondary antibody conjugated to horseradish peroxidase (1:10,000; Sigma-Aldrich) for 1 h at RT. Membranes were incubated with SuperSignal™ West Femto Maximum Sensitivity Substrate (Thermo Fisher Scientific) for 5 min at RT. Membranes were stripped using mild stripping buffer (SDS/glycine; pH 2.2) twice for 10 min, followed by washes with PBS 1X and TBS 1X twice for 5 min each. After stripping and blocking, membranes were re-probed with a different primary antibody. Images were acquired and analyzed using a Gel Doc XR + with ImageLab™software (Bio-Rad). The volume intensity of the CB2R band was normalized against the respective volume intensity of the GAPDH band.

### HIV-1 p24 and total CATB levels

We quantified HIV-1 p24 antigen levels in HIV/MDM supernatants of days 3, 6, 9, and 12pi, using ELISA (Express Biotech International), following manufacturer instructions. Total CATB levels were determined from MDM supernatants at days 3, 6, 9, and 12pi using ELISA (R&D Systems), following manufacturer instructions. The optical densities of HIV-1 p24 and total CATB were determined by photometry using a Varioskan Flash microplate reader (Thermo Fisher Scientific).

### Neuronal apoptosis

Neuronal apoptosis was determined using In Situ Cell Death Detection Kit, which is also known as TUNEL (TdT-mediated dUTP-X nick end labeling) (ROCHE®), as previously described by our laboratory^5^. In summary, HTB-11 cells were rinsed with PBS 1X and exposed to MCM diluted 1:4 in plain EMEM with and without a CATB inhibitor, CA074 (Sigma-Aldrich, 10 µM). Then, HTB-11 were washed twice with PBS 1X and fixed with 4% PFA for 1 h. Fixed neurons were permeabilized with 0.1% TritonX-100 in PBS 1X for 10 min on ice. Neurons were incubated with a TUNEL reaction mix for 1 h in a dark humidity chamber at 37 °C. Neurons were washed three times with PBS 1X and covered with VECTASHIELD® Antifade Mounting Media with DAPI (Vector Laboratories). As a positive control, we incubated neurons with recombinant DNase I (30 U/Ml) for 10 min at RT to induce DNA breaks. As a negative control, we incubated neurons with the label solution without the TdT enzyme. Fluorescence microscopy images were acquired using a Nikon Eclipse E400 microscope, containing a camera SPOT Insight QE and Fluorescence X-cite Series 120. The excitation wavelengths were 405 nm and 488 nm for visualization of total nuclei and apoptotic nuclei, respectively. The magnification was set at 20X. At least three random pictures were acquired and analyzed per condition. The number of apoptotic nuclei was counted and divided by the number of total nuclei, and the percentage of apoptosis was determined. Quantifications and analyses were performed using the NIS Elements Analysis software (Nikon).

### Statistical analysis

We used Graph Pad Prism 6.0 software for statistical analyses. Prior to the comparison of groups, normal distribution was tested. For the analysis of CATB levels between women and men donors, the Mann Whitney test was used. For analysis of CB2R levels, we used two-tailed Wilcoxon-rank tests for single comparisons in Flow Cytometry and Western Blot results and Two-way ANOVA with Tukey’s multiple comparisons test for temporal expression. For analyses of cell viability, we used Two-way ANOVA, and Sidak’s multiple comparisons test or Friedman with Dunn’s multiple comparisons test against NT controls. To analyze the differences in neuronal apoptosis between the groups, we used One-way ANOVA with Tukey’s multiple comparisons test. For the analysis of HIV-1 p24 and CATB levels in response to treatments, we used paired or unpaired t-tests or Wilcoxon-rank tests, when appropriate, to compare each treatment concentration against its vehicle control at each time-point or the uninfected control vs. the HIV-infected control. For co-administration experiments, Friedman with Dunn’s post-test was used. A significant difference was considered at *p* ≤ 0.05.

## Results

### HIV-infected MDM maintains CB2R expression at day 12pi

**Figure 1:**
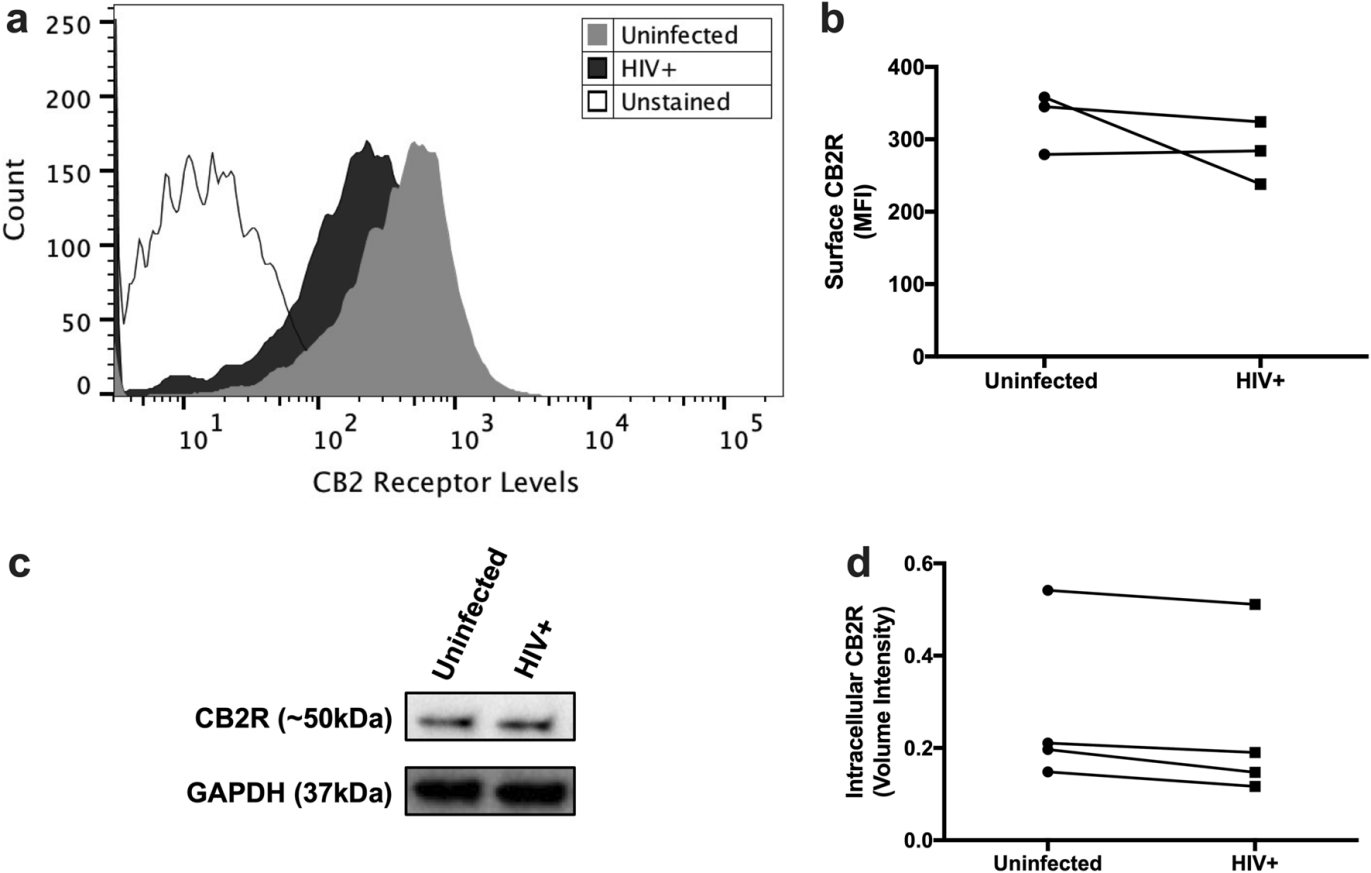
HIV-infected MDM keep expressing surface CB2R at day 12pi. MDM were labeled with a fluorescent CB2R monoclonal antibody (FL4 channel) conjugated with Alexa Fluor® 647 and then analyzed by flow cytometry. (**a**) Representative histograms of extracellular staining show significant differences in fluorescence intensities from HIV + (dark histogram) and uninfected (grey histogram) on the expression of CB2R compared with the nonfluorescent MDM (unshaded histograms to the left. (**b**) A graphic representation of surface CB2R levels per donor is shown. Panels a and b are representative of three different donors (n = 3). An unstained control was included as a technical negative control and is representative of 1 donor (n = 1). (**c**) Representative images of intracellular levels of CB2R and GAPDH expression using Western Blot. Images were cropped from the same blot after stripping and reprobing with a different antibody and were acquired using different exposure times. Full-length blots are presented in Supplementary Fig. 4 (**d**) A graphic representation of intracellular CB2R levels per donor is shown. Figures c and d are representative of four different donors (n = 4).

Since MDM were exposed to CB2R agonists until day 12pi, we wanted to determine if CB2R was still being expressed at this endpoint. MDM from day 12pi were detached from culture flasks, stained with anti-human CB2R antibody, and levels of CB2R were quantified using Flow Cytometry. Surface CB2R expression was observed in both uninfected and HIV-infected MDM at day 12pi (Fig. 1a). However, no significant differences in CB2R levels were observed between both groups (*p* = 0.5000) (Fig. 1b). These results correlated with intracellular CB2R expression levels measured by western blot, where no significant differences were observed between the groups (*p* = 0.1250) (Fig. 1c,d). These results indicate that HIV-infected MDM keep expressing surface and intracellular CB2R at day 12pi. However, donor-to-donor differences in CB2R expression levels were observed.

### CB2R agonists decrease HIV-1 replication and CATB secretion from MDM

**Figure 2:**
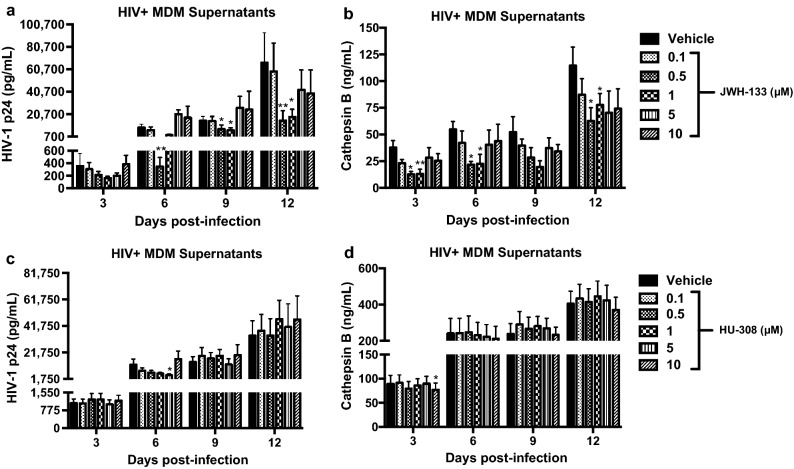
CB2R agonists treatment decrease HIV-1 replication and CATB secretion from MDM. MDM were infected with HIV-1 and treated with CB2R agonists, JWH-133 and HU-308. HIV-1 p24 and CATB levels were measured from supernatants of days 3, 6, 9, and 12pi by ELISA. (**a**) Temporal HIV-1 p24 levels in supernatants of HIV-infected MDM treated with JWH-133. (**b**) Temporal CATB secretion levels in supernatants of HIV-infected MDM treated with JWH-133. Figures a and b are representative of at least four different donors (n = at least 4). (**c**) Temporal HIV-1 p24 levels in supernatants of HIV-infected MDM treated with HU-308. (**d**) Temporal CATB secretion levels in supernatants of HIV-infected MDM treated with HU-308. Figures c and d are representative of at least six different donors (n = at least 6). Graphs are presented using the mean and the ± standard error of the mean (SEM). *p < 0.05, **p < 0.01 vs. vehicle control at its respective time-point.

Previous studies from our laboratory have demonstrated that increased CATB secretion from HIV-infected MDM at day 12pi leads to neurotoxicity^5,7–10^. Therefore, HIV-infected MDM were treated with CB2R selective agonists JWH-133 or HU-308 at five different concentrations (0.1, 0.5, 1, 5, and 10 µM), and treatments were maintained until day 12pi. On day 12dpi, media was removed, and MDM were exposed to untreated serum-free media for 24 h to collect MCM at 13dpi for the neuronal apoptosis assay. Cell viability was assessed at the end of cultures (13dpi). None of the concentrations of CB2R agonists had a significant effect on HIV-infected MDM viability (Supplementary Fig. 2). JWH-133 significantly decreased HIV-1 p24 levels at days 6, 9, and 12pi in a dose-dependent manner (Fig. 2a). JWH-133 significantly decreased CATB levels as early as 3dpi at concentrations of 0.5 µM (*p* = 0.0248) and 1 µM (*p* = 0.0071), and this effect was sustained until day 12pi (Fig. 2b). A visual dose-dependent decrease in CATB levels was observed at day 9pi; however, it did not reach statistical significance at any of the concentrations. On the other hand, HU-308 decreased HIV-1 p24 levels at day 6pi at a concentration of 5 µM (*p* = 0.0174), but this effect was lost over time (Fig. 2c). Additionally, HU-308 decreased CATB levels at day 3pi at a concentration of 10 µM (*p* = 0.0242), but this effect was lost over time (Fig. 2d). These results suggest that there are agonist-specific effects on HIV-1 replication and CATB secretion.

In our experiments, we observed MDM cultures with increased (*p* = 0.0209) or decreased (p = 0.0299) CATB secretion after HIV infection at day 12pi compared to uninfected controls (Supplementary Fig. 3). In a recent study, we demonstrated that CATB secretion from HIV-infected MDM was associated with oxidative stress^9^. Additionally, in recent studies we have demonstrated that MDM cultures with increased CATB secretion after HIV infection induce significant neuronal apoptosis, whereas MDM cultures with decreased CATB secretion after HIV infection fails to induce significant neuronal apoptosis (Zenon et al., in press). Thus, we analyzed the effect of JWH-133 in MDM cultures with increased CATB secretion. Results show that in MDM with increased CATB secretion after HIV infection (*p *= 0.0209), JWH-133 treatment at 0.5 µM (*p* = 0.0387) and 1 µM (*p* = 0.0159) showed a significant decrease in total CATB levels in comparison to HIV-infected untreated control (Supplementary Fig. 3a). The JWH-133 agonist at 0.5 µM was selected for the next experiments because this was the minimum concentration that showed a significant decrease in CATB and HIV-1 p24 levels at day 12pi. In summary, these results suggest that the JWH-133 agonist was more effective than HU-308 in decreasing HIV-1 p24 and CATB levels in a dose and time-dependent manner”.

### JWH-133 prevents HIV-induced increase in surface CB2R expression and induces oscillating expressions over time.

**Figure 3:**
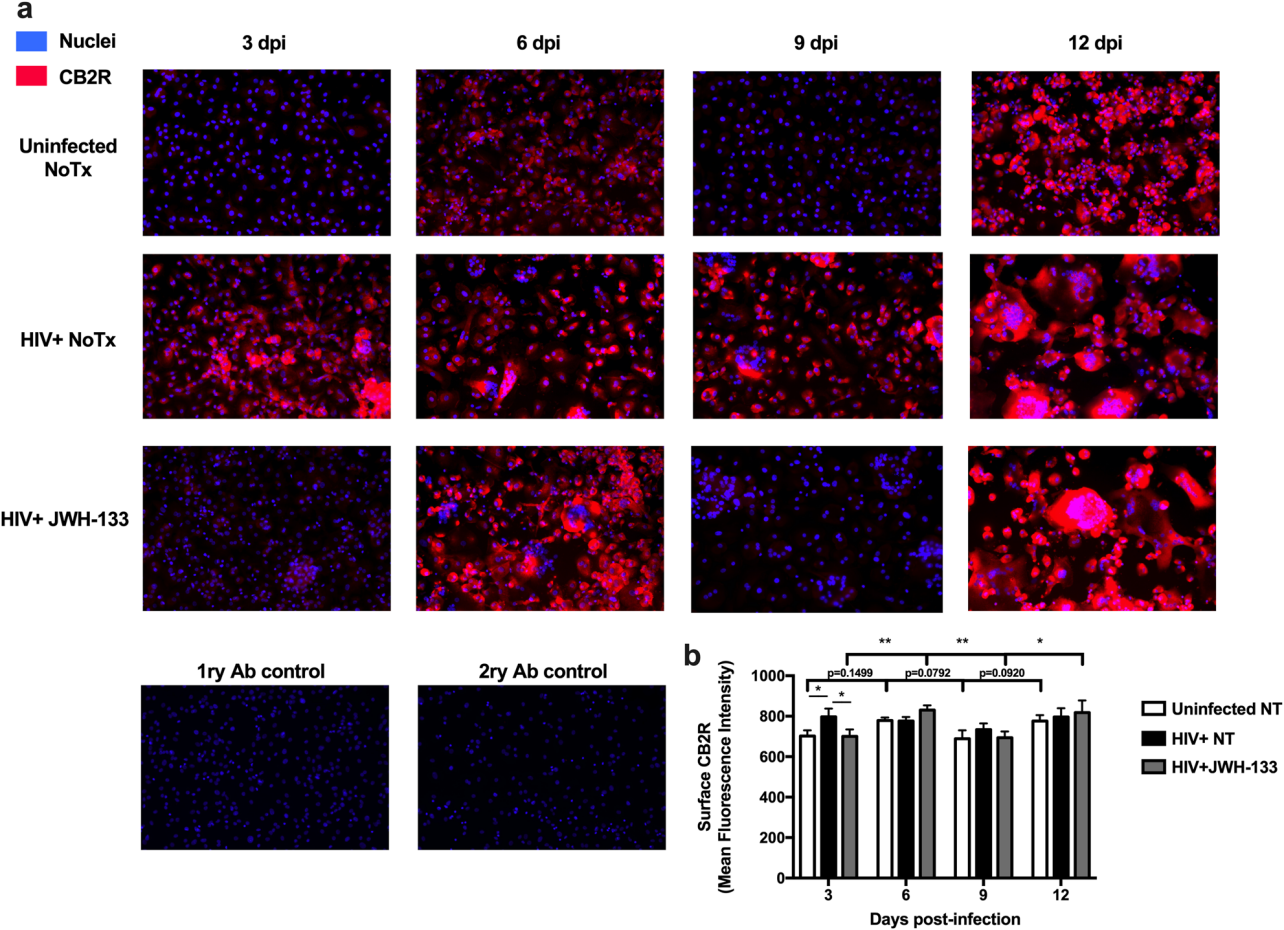
JWH-133 prevents HIV-induced increase in surface CB2R expression and induces oscillating expressions over time. MDM were cultured in 8-well chamber slides, infected with HIV-1_ADA_, and treated with JWH-133 at 0.5 µM. Slides were fixed at days 3, 6, 9, and 12dpi. Cells were stained with an anti-CB2R antibody (red) and nuclei were stained with DAPI (blue). At least 3 different random pictures per condition were acquired. Representative immunofluorescence images are shown. Graphs are presented using the mean ± standard error of the mean (SEM). This figure is representative of three different donors (n = 3). *p < 0.05, **p < 0.01.

We analyzed the temporal surface expression of CB2R in HIV-infected MDM in the absence or presence of JWH-133 treatment at 0.5 µM. Results showed that HIV infection increased surface CB2R expression at day 3pi (*p* = 0.0357) (Fig. 3). A visual increase was observed in HIV-infected MDM at day 9pi compared to uninfected controls; however, it did not reach statistical significance (*p* = 0.3948). Treatment with JWH-133 prevented the HIV-induced increase in surface CB2R expression levels at day 3pi (*p* = 0.0332). In terms of differences in temporal expression, uninfected MDM showed trends towards significant oscillations over time, as shown in the graph. HIV-infected MDM showed no significant changes in CB2R expression over time, and JWH-133 treatment induced significant oscillations of CB2R expression in HIV-infected MDM over time (3dpi vs. 6dpi: *p* = 0.0097; 6dpi vs. 9dpi: *p* = 0.0067; 9dpi vs. 12dpi: *p* = 0.0131). These results suggest that HIV-1 infection increases surface CB2R expression in a time-dependent manner, and this effect is prevented by JWH-133 treatment. Also, JWH-133 treatment induces significant CB2R expression oscillations over time.

### JWH-133 decreases HIV-1 replication and CATB secretion through CB2R activation

**Figure 4:**
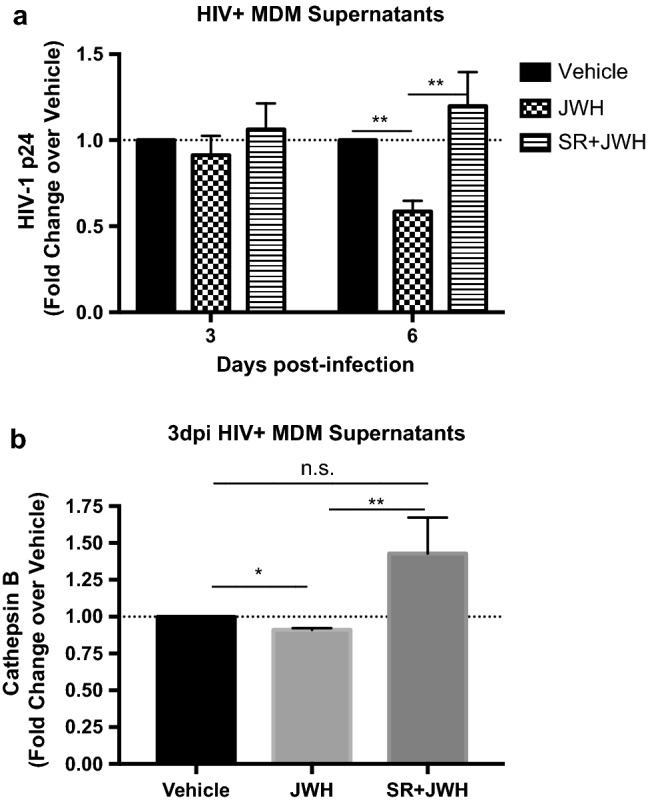
JWH-133 decreases HIV-1 replication and CATB secretion through CB2R activation. MDM were cultured in 24-wells plates and infected with HIV-1_ADA_. After removal of residual virus, cells were treated with CB2R antagonist SR144528 (SR: 1 µM) for 1 h, followed by JWH-133 (JWH) treatment at 0.5 µM. Treatments were maintained for 6dpi, exchanging half of the media at day 3pi and repeating the co-administration protocol. HIV-1 p24 and CATB levels were measured in HIV-infected MDM supernatants using ELISA. HIV-1 p24 and CATB levels were normalized against its vehicle control per donor. (**a**) HIV-1 p24 levels in HIV-infected MDM after co-administration of CB2R ligands. This figure is representative of at least eight different donors (n = 8). (**b**) CATB levels in HIV-infected MDM after co-administration of CB2R ligands. This figure is representative of ten different donors (n = 10). Graphs are presented using the mean ± standard error of the mean (SEM). *p < 0.05, **p < 0.01.

To determine if JWH-133 decreases HIV-1 replication and CATB secretion through CB2R activation, after removal of residual virus, MDM were incubated with a CB2R antagonist SR144528 at 1 µM for 1 h before JWH-133 treatment at 0.5 µM. On day 3pi, half of the media was collected, and co-administration of ligands was repeated and maintained until day 6pi. Supernatants from days 3pi and 6pi were used for the determination of HIV-1 p24 and total CATB levels. These time-points were selected because 3dpi was the minimum day at which a significant decrease in CATB levels was observed following JWH-133 treatment (Fig. 2b), and day 6pi was the minimum day at which the effect in decreasing HIV-1 p24 was observed (Fig. 2a). Results showed that JWH-133 decreased HIV-1 p24 levels at day 6pi (*p* = 0.0050), and pre-treatment with SR144528 reversed this effect (*p* = 0.0075) (Fig. 4a). JWH-133 decreased CATB levels at day 3pi (*p* = 0.0139) and pre-treatment with SR144528 at 1 µM reversed JWH-133 effect (*p* = 0.0037) (Fig. 4b). These results suggest that JWH-133 decreases HIV-1 replication and CATB secretion through CB2R activation. Results from HIV-1 replication are consistent with previous studies that determined that JWH-133 decreases HIV-1 replication from primary MDM through CB2R activation.^23^

### JWH-133 decreases HIV/MDM-induced neuronal apoptosis and CATB neurotoxic potential

**Figure 5:**
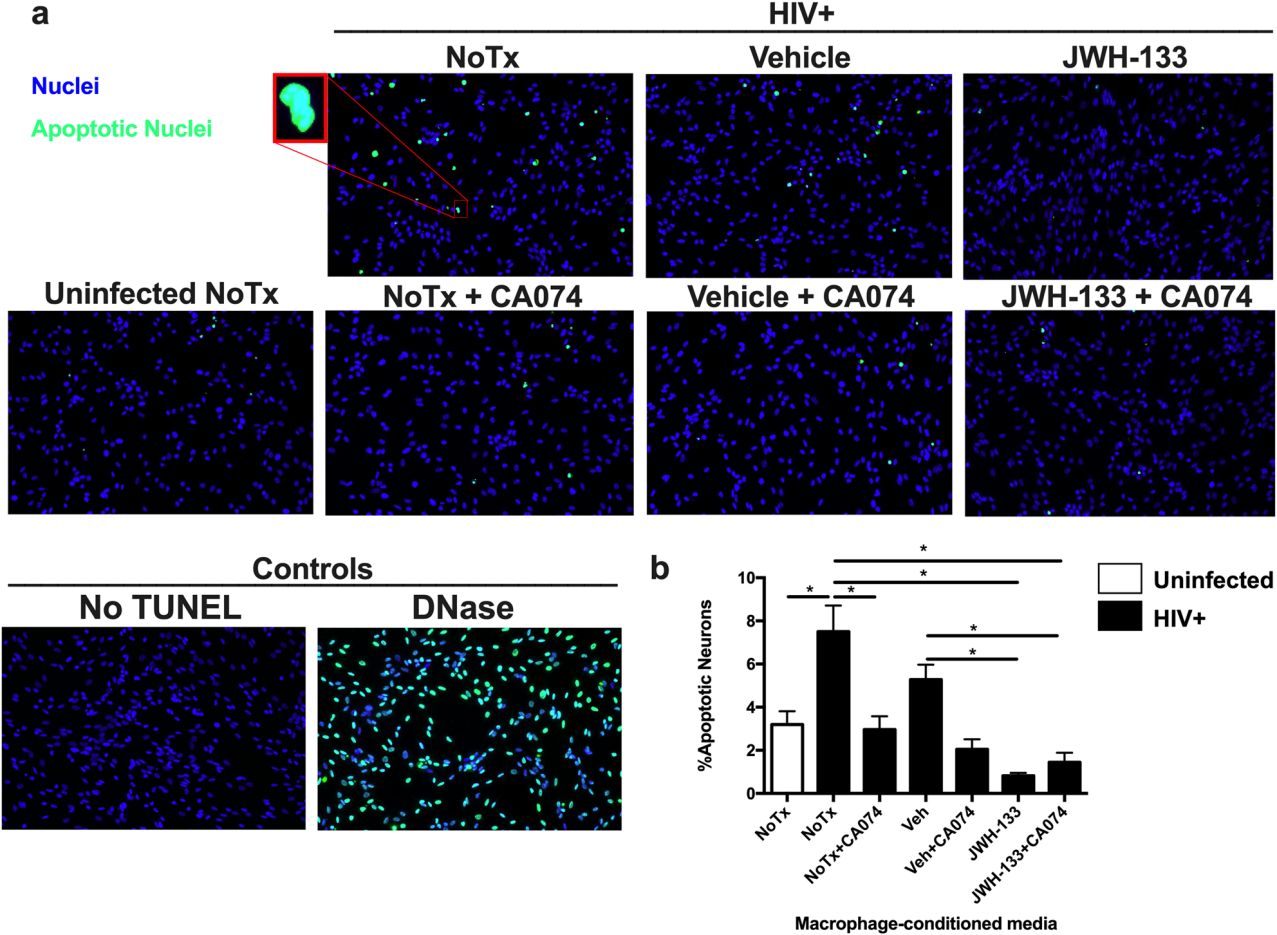
JWH-133 decreases HIV/MDM-induced neuronal apoptosis and CATB neurotoxic potential. Uninfected and HIV-infected MDM were untreated or treated with vehicle and JWH-133 (0.5 µM) for 12dpi. On day 13pi, MCM from donors that had increased CATB secretion after HIV-1 infection compared to the uninfected control were used to determine the effect of JWH-133 treatment in HIV/MDM-induced neuronal apoptosis and CATB neurotoxic potential. (**a**) Representative immunofluorescence images of human neuroblastoma cells that were exposed to MCM in the presence or absence of the CATB inhibitor, CA074. Cyan fluorescence indicates TUNEL positive cells (apoptotic neurons), whereas nuclei stained with DAPI are shown in blue. DNase and No TUNEL were included as positive and negative controls, respectively. (**b**) Cyan and blue nuclei were counted in at least 3 random fields per condition and results are shown as %Apoptotic Neurons. This figure is representative of four different donors (n = 4). Graphs are presented using the mean and the ± standard error of the mean (SEM). *p < 0.05.

Previous studies in our laboratory have demonstrated that MCM from HIV-infected MDM with increased CATB levels induces significant neuronal apoptosis compared to uninfected controls^5,7–10^. This effect is reversed by the addition of a CATB inhibitor (CA074) or a CATB antibody to the MCM of HIV-infected MDM^5,7–10^. Thus, MCM from HIV-infected MDM cultures with increased CATB levels compared to uninfected controls (Supplementary Fig. 3a) were used to determine the effect of JWH-133 on HIV/MDM-induced neuronal apoptosis and CATB neurotoxic potential. Neuroblastoma cultures were exposed to MCM from HIV-infected MDM for 24 h in the presence or absence of CA074. Neuronal apoptosis was assessed by TUNEL assay. MCM from HIV-infected MDM that were treated with JWH-133 significantly decreased the percentage of apoptotic neurons compared to MCM from HIV-infected untreated (*p* = 0.0468) and vehicle-treated (*p* = 0.0391) MDM (Fig. 5). A significant reduction in neuronal apoptosis was observed in the JWH-133 + CA074 condition compared to MCM from HIV-infected MDM that were treated with vehicle (*p* = 0.0409) or untreated (*p* = 0.0252). However, no significant differences were observed between JWH-133 and JWH-133 + CA074 MCM (*p* = 0.5669), suggesting that treating HIV-infected MDM with JWH-133 is sufficient to decrease CATB-mediated neurotoxicity to a maximum level. Our results suggest that JWH-133 decreases HIV/MDM-induced neuronal apoptosis and CATB neurotoxic potential.

## Discussion

In this study, we demonstrated that CB2R agonist JWH-133 decreased HIV/MDM-induced neuronal death and CATB neurotoxic potential by decreasing CATB secretion from HIV-infected MDM. The mechanism by which JWH-133 decreased CATB secretion was through CB2R activation. JWH-133 decreased HIV-1 replication in MDM through CB2R activation by day 6pi. These results are consistent with previous studies that have demonstrated that JWH-133 treatment decreases HIV-1 replication after day 4pi^24,36^. CB2R agonists, JWH-133 and HU-308, decreased HIV-1 replication and CATB secretion from HIV-infected MDM in a time and dose-dependent manner. However, JWH-133 was more effective than HU-308 at maintaining a chronic downregulation of HIV-1 replication and CATB secretion in MDM. We hypothesize that continuous administration of HU-308 leads to CB2R desensitization in HIV-infected MDM through an unknown agonist-specific mechanism. Further studies are needed to confirm these hypotheses.

This is the first study that evaluates the effect of CB2R agonists against HIV/MDM-induced neuronal death. Additionally, this is the first study that evaluates temporal surface CB2R expression in HIV-infected MDM in the presence or absence of a CB2R agonist. We demonstrated that HIV-infected MDM increases surface CB2R expression at day 3pi and that it is prevented by JWH-133 treatment. Similar results were observed at day 9dpi; however, not to a significant level. This HIV-induced increase in surface CB2R levels is consistent with a previous study in primary HIV-infected MDM, which reported a 2.28-fold increase in surface CB2R levels over uninfected MDM at day 7pi^24^. These results are supported by other studies that have demonstrated a time-dependent increase in CB2R expression in macrophages following an inflammatory insult^45–47^. JWH-133 induced significant oscillations of surface CB2R levels over time. These results suggest that there is a time-dependent regulation of surface CB2R expression after HIV-1 infection of MDM and JWH-133 treatment. We hypothesize that constitutive activity, internalization, and recycling of CB2R in the absence of JWH-133 is responsible for the trends in surface expression oscillations in uninfected MDM, as previous studies have demonstrated in HEK-293 stably expressing CB2R^48^. On the other hand, we speculate that HIV-1 is preventing the constitutive internalization of CB2R by an unknown mechanism. However, after JWH-133 treatment, agonist-induced internalizations and recycling could explain the significant oscillations observed in this group. This hypothesis was formulated based on previous studies that have demonstrated that CB2R can be recycled back to the surface after agonist-induced internalization^48,49^. Further in vitro and in vivo studies on CB2R internalization and recycling are needed to evaluate these hypotheses.

We hypothesize that the intracellular mechanism by which JWH-133 decreases CATB secretion from HIV-infected macrophages after activation of CB2R is by reducing oxidative stress. This hypothesis is formulated based on a recent study in our laboratory in which we demonstrated that dimethyl fumarate, an antioxidant, decreases oxidative stress and CATB secretion from HIV-infected MDM^9^. Moreover, CB2R activation inhibits oxidative stress in macrophages^50–52^. Since CATB plays a significant role in HIV/MDM-induced neuronal death, the decreased CATB levels after JWH-133 treatment could have led to a decreased neurotoxicity. Other studies have focused on the effect of CB2R ligands against the neurotoxicity of HIV-1 viral proteins. These studies demonstrated protective effects of CB2R activation against HIV-1 gp120-induced synapse loss, impaired neurogenesis, and neuronal death^37–40^. Also, a recent study demonstrated that CB2R activation protects from Tat-induced neuronal degeneration and death following fatty acid amide hydrolase inhibition, an enzyme that metabolizes our main endocannabinoid anandamide^41^. However, other studies have not found neuroprotective effects of CB2R activation against GABAergic neurotransmission impairment, excitability, and death^53,54^.

Current studies in our laboratory are focused on understanding the mechanisms associated with CATB secretion from macrophages as well as the mechanisms of its neurotoxicity. Future studies will explain if JWH-133 decreases CATB secretion by reducing oxidative stress. HIV-1_ADA_ CCR5-tropic strain was selected for this study as it is a macrophage-tropic strain that has been used in all our previous studies that demonstrate increased CATB secretion and neurotoxicity^5,7–10^. Highly cytopathic CXCR4-tropic virus strains were not considered for these studies because a recent study demonstrated that HIV-1 infection of MDM with CXCR4-tropic virus induces productive HIV-1 replication in MDM until day 7pi, and then it decreases drastically until it is not detected at day 10pi due to viral-induced cell death^55^. Thus, we hypothesize that highly cytopathic CXCR4-tropic virus will not allow us to maintain sufficient viable macrophages in culture until the time-point where HIV-infected macrophages induce CATB-induced neuronal death (day 12pi), as demonstrated by previous studies in our laboratory^5^.

In summary, CB2R activation reduces HIV-1 replication and CATB secretion from HIV-infected MDM. However, differences between agonists in terms of time and effective concentration were observed. JWH-133 decreased HIV/MDM-induced neuronal death and CATB neurotoxic potential. These results are important because they reveal new aspects of CB2R ligands that contribute to the development of effective strategies against HAND. In conclusion, our findings suggest that CB2R agonists represent a potential therapeutic strategy against HIV/MDM-induced neuronal death. However, long-term in vivo studies are needed to validate these in vitro findings and evaluate the effectiveness of CB2R agonists against HIV-induced neurotoxicity and HAND.

## Supplementary Information

Supplementary Information.

## Data Availability

Most of the data generated or analyzed during this study are included in this published article. All datasets generated during and/or analyzed in the current study are available from the corresponding author on reasonable request.
