# Supplementary material for: Cannabinoid receptor type 2 agonist JWH-133 decreases cathepsin B secretion and neurotoxicity from HIV-infected macrophages
Source: Sci Rep. 2022 Jan 7;12:233. doi: 10.1038/s41598-021-03896-3 (PMC8741953; doi:10.1038/s41598-021-03896-3)
Supplement: Supplementary file 1 — Supplementary Information. [file 41598_2021_3896_MOESM1_ESM.docx]

**Supplementary Fig. 1 HIV-infected MDM from women secrete higher levels of CATB compared to men.** PBMCs from 7 healthy women (n=7) and 5 healthy men (n=5) were cultured in T25 flasks at a concentration of 10x10^6^ cells/flask. MDM were infected with HIV-1_ADA_ and maintained in culture exchanging half of the media every three days, as previously described in Methods. CATB levels were measured from HIV-infected MDM supernatants. Graphs are presented using the mean and the ± standard error of the mean (SEM). **p<0.01

**Supplementary Fig. 2 Chronic CB2R ligands treatments do not affect cell viability of HIV-infected MDM.** MDM were infected with HIV-1 _ADA_ and treated with CB2R ligands (a) JWH-133, (b) HU-308, and (c) SR144528, as previously described in Methods. As a positive control, we incubated untreated MDM with 1% Triton X-100 for 24h prior to the end of cultures. Cell viability was assessed using an MTT assay. Graphs are presented using the mean and the ± standard error of the mean (SEM). (a) Experimental conditions and negative control (uninfected NT) are representative of at least 5 different donors (n=at least 5); Triton X-100 positive control is representative of three different donors (n=3). (b) This graph is representative of five different donors (n=5). (c) This graph is representative of five different donors (n=5) **p<0.01 vs. uninfected no treatment group (NT), ***p<0.001 vs. HIV+ NT, ****p<0.0001 vs. uninfected NT.

**Supplementary Fig. 3 JWH-133 decreases CATB secretion in donors with increased CATB levels after HIV infection at 12dpi.** MDM were infected with HIV-1 _ADA_ and treated with different concentrations of JWH-133, as previously described in Material and Methods. (a) Donors with increased cathepsin B secretion after HIV-1 infection are shown. MCM from these donors were used for the neuronal apoptosis assay. Graphs are presented using the mean and the ± standard error of the mean (SEM). This figure is representative of at least four different donors (n=at least 4). (b) Donors with decreased cathepsin B secretion after HIV-1 infection are shown. Experimental conditions (JWH-133 at 0.1 µM, 0.5 µM, and 1 µM) and uninfected and HIV+ controls are representative of four different donors (n=4); whereas experimental conditions of JWH-133 at 5 µM and 10 µM are representative of two (n=2) different donors and were not considered for statistical analyses. *p<0.05

**Supplementary Fig. 4 Intracellular Expression of CB2R in MDM at day 12pi.** Uninfected and HIV-infected MDM were cultured until day 12pi as previously described in Materials and Methods section. Whole cell lysates were collected at day 12pi and intracellular CB2R expression was quantified using Western Blot, using GAPDH as a loading control. Images were analyzed using Image Lab software. All pictures were merged with a colorimetric picture of the ladder run in the same gel. a) Picture of blot showing CB2R expression at ~50kDa (70 seconds of exposure at Chemi Hi resolution). b) Picture of blot showing GAPDH expression at ~37kDa (20 seconds of exposure at Chemi Hi resolution). (1) Ladder, (2) Blank, (3) Negative Control=SK-N-SH cell line, (4) Blank, (5) Donor (D)1-Uninfected, (6) D1- HIV+, (7) D2-Uninfected, (8) D2-HIV+, (9) D3-Uninfected, (10) D3-HIV+, (11) D4-Uninfected, (12) D4-HIV+(nonproductive HIV-1 replication control), (13) D5-Uninfected, (14) D5-HIV+, (15) Negative Control=HEK293T cell line. Samples from D4 were not considered for statistical analyses as this donor had nonproductive HIV-1 replication by the end of cultures (as determined by measuring HIV-1 p24 levels in supernatants) and was included here as a control. (c and e) Same blot showing CB2R expression after 40 and 10 secs of exposure, respectively. (d and f) Same blot showing GAPDH expression after 10 and 2 secs of exposure, respectively. (g and h) Colorimetric pictures of ladder used for merging pictures of CB2R and GAPDH expression, respectively. Cropped images shown in Fig.1c come from blots a and b.
